# Parks

**Published:** 1870-09

**Authors:** 


### PARKS.

THE Central Park of New York contains, in round numbers, 850 acres, and has cost ten millions of dollars; but the increased value of property around this park yields a tax which more than pays the interest on the outlay. Five million persons visit the park every year. The Prospect Park of Brooklyn contains 578 acres, and has cost already seven millions of dollars.

There is another park, common to New York and Brooklyn, which many visit; some go there to stay — forever! It is the beautiful, the magnificent

### GREENWOOD CEMETERY.

One hundred and thirty-seven thousand bodies are buried there, of which seven thousand five hundred were interred during 1869

New York City has an area of twenty-two square miles, or fourteen thousand acres of land.

1,000,000 persons live in New York,
25,000 die every year,
31,000 are born,
17,000 are married yearly; one half of all the inhabitants of New York City were born in foreign lands.

Twenty-five million letters and newspapers are delivered to New Yorkers at their dwellings every year, being twenty-five to each individual. There are 140,000 lots in New York, about one half of which are built upon. One vacant lot on Fifth Avenue, corner of Forty-third Street, twenty-five feet front, extending back a hundred feet, is valued at seventy thousand dollars; the owner says if he cannot get that price for it, it can stay there.

We had rather live in New York City than in any other spot on the face of the globe; and many thousands would like to live there too, but there are only two hundred thousand names in the Directory, and of these only six hundred and sixty-six (666) are living in Fifth Avenue. There are very many persons who consider a residence in Fifth Avenue as one of the greatest of all human privileges; but its noise and dust so greatly detract from its desirableness that it is rapidly filling up, in its lower third, with business houses and hotels; in fact, there are but few private residences in the Avenue, below Twenty-sixth Street, which is nearly half way to the entrance of the Central Park at Fifty-ninth Street.

By the aid of its avenues and parks, and with the advantage of being on a ridge of land descending on both sides to the river, New York is capable of being made the healthiest city on the globe. Fifth Avenue being on the ridge is the healthiest street; but Madison Avenue, as to cleanliness, quiet, and comfort, with its spacious and elegant mansions, is preferred by many. Still, Fifth Avenue above Thirty-second Street, is likely to be considered the "West End," up to the Central Park entrance, for a long time to come, for it is the grandest city street in the world, considering its extent.

